# Assessing cell lines with inducible depletion of cohesin and condensins components through analysis of metaphase chromosome morphology

**DOI:** 10.18699/vjgb-24-16

**Published:** 2024-04

**Authors:** A.M. Yunusova, A.V. Smirnov, I.E. Pristyazhnuk, T.A. Shnaider, E.K. Maltseva, S.D. Afonnikova, O.A. Gusev, N.R. Battulin

**Affiliations:** Institute of Cytology and Genetics of the Siberian Branch of the Russian Academy of Sciences, Novosibirsk, Russia; Institute of Cytology and Genetics of the Siberian Branch of the Russian Academy of Sciences, Novosibirsk, Russia; Institute of Cytology and Genetics of the Siberian Branch of the Russian Academy of Sciences, Novosibirsk, Russia; Institute of Cytology and Genetics of the Siberian Branch of the Russian Academy of Sciences, Novosibirsk, Russia; Novosibirsk State University, Novosibirsk, Russia; Institute of Cytology and Genetics of the Siberian Branch of the Russian Academy of Sciences, Novosibirsk, Russia; Life Improvement by Future Technologies (LIFT) Center, Moscow, Russia Kazan Federal University, Kazan, Russia Endocrinology Research Center, Moscow, Russia; Institute of Cytology and Genetics of the Siberian Branch of the Russian Academy of Sciences, Novosibirsk, Russia Novosibirsk State University, Novosibirsk, Russia

**Keywords:** SMC proteins, degron, chromosome condensation, SMC белки, дегрон, конденсация хромосом

## Abstract

One of the most productive strategies for finding the functions of proteins is to study the consequences of loss of protein function. For this purpose, cells or organisms with a knockout of the gene encoding the protein of interest are obtained. However, many proteins perform important functions and cells or organisms could suddenly lose fitness when the function of a protein is lost. For such proteins, the most productive strategy is to use inducible protein degradation systems. A system of auxin-dependent protein degradation is often implemented. To use this system, it is sufficient to introduce a transgene encoding a plant-derived auxin-dependent ubiquitin ligase into mammalian cells and insert a sequence encoding a degron domain into the gene of interest. A crucial aspect of development of cell lines engineered for inducible protein depletion is the selection of cell clones with efficient auxin-dependent degradation of the protein of interest. To select clones induced by depletion of the architectural chromatin proteins RAD21 (a component of the cohesin complex) and SMC2 (a component of the condensin complex), we propose to use the morphology of metaphase chromosomes as a convenient functional test. In this work, we obtained a series of clones of human HAP1 cells carrying the necessary genetic constructs for inducible depletion of RAD21 and SMC2. The degradation efficiency of the protein of interest was assessed by flow cytometry, Western blotting and metaphase chromosome morphology test. Based on our tests, we showed that the clones we established with the SMC2 degron effectively and completely lose protein function when induced by auxin. However, none of the HAP1 clones we created with the RAD21 degron showed complete loss of RAD21 function upon induction of degradation by auxin. In addition, some clones showed evidence of loss of RAD21 function even in the absence of induction. The chromosome morphology test turned out to be a convenient and informative method for clone selection. The results of this test are in good agreement with flow cytometry analysis and Western blotting data.

## Abstract (ru)

Одна из самых продуктивных стратегий поиска функций различных белков – исследование последствий потери функции белка. Часто для этого получают клетки или организмы с нокаутом гена, кодирующего белок интереса. Однако многие белки выполняют настолько важные функции, что клетка или организм резко теряют жизнеспособность при потере функции такого белка. Для этих белков наиболее продуктивной стратегией является применение систем индуцируемой деградации белка. Часто используют систему ауксин - зависимой деградации белков. Для применения этой системы достаточно ввести в клетки млекопитающих трансген, кодирующий растительную ауксин-зависимую убиквитин лигазу, и включить в ген интереса последовательность, кодирующую дегроновый домен. Важный этап создания клеток, способных к индуцируемой деплеции белка, – отбор клеточных клонов с эффективной ауксин-зависимой деградацией белка интереса. Для отбора клонов с индуцируемой деплецией архитектурных белков хроматина RAD21 (компонент когезинового комплекса) и SMC2 (компонент конденсинового комплекса) мы предлагаем использовать морфологию метафазных хромосом как удобный функциональный тест. В данной работе мы получили серию клонов клеток человека HAP1, несущих необходимые генетические конструкции для индуцируемой деплеции RAD21 и SMC2. Эффективность деградации белка интереса была оценена с помощью проточной цитофлуориметрии, Вестерн-блоттинга и теста на морфологию метафазных хромосом. На основе проведенных тестов мы продемонстрировали, что созданные нами клоны с дегроном SMC2 эффективно и полно теряют функцию белка при индукции ауксином. При этом ни один из созданных нами клонов HAP1 с дегроном RAD21 не показал полной потери функции RAD21 при индукции деградации ауксином. Кроме того, некоторые клоны имели признаки потери функции RAD21 даже в отсутствие индукции. Использованный нами тест на морфологию хромосом оказался удобным и информативным для отбора клонов. Результаты этого теста хорошо согласуются с данными проточной цитофлуориметрии анализа и Вестерн-блоттинга.

## Introduction

Chromatin architectural proteins play a crucial role in maintaining the three-dimensional structure of the genome (Kabirova et al., 2023). Among them, special attention is drawn to the cohesin and condensin complexes belonging to the SMC (structural maintenance of chromosomes) family of proteins. Cohesin has many different functions: it ensures cohesion of sister chromatids after replication (Losada et al., 1998), forms loops and TADs (topologically associating domains) via the loop extrusion mechanism (Nuebler et al., 2018), and is involved in the repair of DNA breaks (Litwin et al., 2018). Condensins, on the other hand, organize the loops of metaphase chromosomes during cell division (Gibcus et al., 2018). These functions can be considered critical for maintaining cell life; therefore, homozygous loss-of-function mutations in the genes encoding cohesin subunits are lethal for cells

The inability to obtain dividing cells without cohesin makes it difficult to study the cohesin complex. To characterize the consequences of cohesin loss, researchers use various tricks. For example, V.C. Seitan et al. utilized a conditional knockout approach to examine the effects of cohesin loss in postmitotic thymocytes in vivo (Seitan et al., 2011). The absence of cell division in mature thymocytes makes them more tolerant to the severe consequences of cohesin loss, such as disruption of mitotic mechanics due to loss of chromatid cohesion. However, for use in cell cultures, conditional knockout of proteins crucial for cell division is almost inapplicable, since cells actively proliferate in culture, and DNA excision of a gene fragment using Cre recombinase in a specific cell rarely occurs. Therefore, it takes a long time (several days or weeks) for a knockout to occur in a significant part of the cells

The study of chromatin architectural proteins has greatly advanced with the development of inducible protein degradation methods. A comprehensive overview of these technologies can be found in the article by E. de Wit and E.P. Nora (2023). Among the various systems used for degrading proteins, the auxin-dependent protein depletion system is currently the most widely employed (Phanindhar, Mishra, 2023). While this system offers great potential for solving scientific problems, its application comes with several challenges that are not adequately addressed in the existing literature.

One of these problems is the selection of clones that carry all the necessary genetic modifications and are truly capable of inducible depletion of the protein of interest. The difficulty is that the addition of the degron tag can negatively affect protein function, which reduces cell fitness. Under such conditions, a selection advantage is given to cells that have somehow blocked the functioning of the introduced system of auxindependent protein degradation, for example, due to epigenetic silencing of exogenous plant ubiquitin ligase (Yunusova et al., 2021). Therefore, clone selection based on Western blotting or loss of protein function tests is of particular importance for the application of auxin degron technology.

Here, we used the involvement of cohesin and crondensins in the formation of the metaphase chromosome to assess the completeness of the loss of protein function in cell clones with inducible depletion of these complexes. We showed that assessment of chromosome morphology is a convenient functional test that allows screening of clones.

## Materials and methods

Cell culture and cell lines. The human HAP1 cell line (a near-haploid cell line derived from the KBM-7 cell line) was purchased from Horizon Discovery. HCT116-based cell lines with auxin-inducible degron-tagged RAD21 and SMC2 genes were kindly provided by Dr. Masato Kanemaki. Cells were maintained at 37 °C in a humidified atmosphere with 5 % CO2 in growth medium that consisted of IMDM supplemented with 10 % FBS (vol/vol), 2 mM GlutaMAX (all from Thermo Fisher Scientific, USA) and 50 U/ml penicillin/50 mg/ml streptomycin (Capricorn Scientific GmbH, Germany). After reaching 70–80 % confluency, cells were detached with 0.05 % trypsin/EDTA and replated at a 1:3 ratio into new cell culture dishes. Cells subjected to flow cytometric analysis were resuspended in PBS. Flow cytometric analysis was performed on a BD FACSAria (BD Biosciences).

Auxin treatment. For inducing degradation of proteins fused with miniIAA7, 500 μM Indole 3 acetic Acid (IAA, I2886, Sigma-Aldrich, USA) was added directly to the culture medium. HCT116 cell lines were treated with 1 μM 5-Ph-IAA at the appropriate time intervals.

Plasmids and constructs. Donor vectors with homology arms, degron tag (miniIAA7-eGFP) and selection cassette were assembled in one reaction using the Gibson Assembly (NEBuilder HiFi DNA Assembly Master Mix, NEB, USA) in the pMK290 backbone (Addgene, 72828). miniIAA7-eGFP fragment was amplified from the vector pSH-EFIRES-BSeipin- miniIAA7-mEGFP (Addgene, 129719). Homology arms for RAD21 (NCBI Entrez Gene ID: 5885) and SMC2 (NCBI Entrez Gene ID: 10592) were PCR-amplified from human genomic DNA with Q5 polymerase (NEB, USA). For auxin receptor F-box protein overexpression AtAFB2 plasmids were used (pSH-EFIRES-P-AtAFB2-mCherry-weak NLS vector (Addgene, 129717)). SgRNA targeting the last codon of the RAD21 and SMC2 genes were cloned into gRNA_Cloning Vector (Addgene, 41824). For CRISPR/Cas9 gene targeting the human codon-optimized Cas9 expression plasmid (Addgene, 41815) was used. The list of primers and gRNA sequences is shown in Table.

**Table 1.**
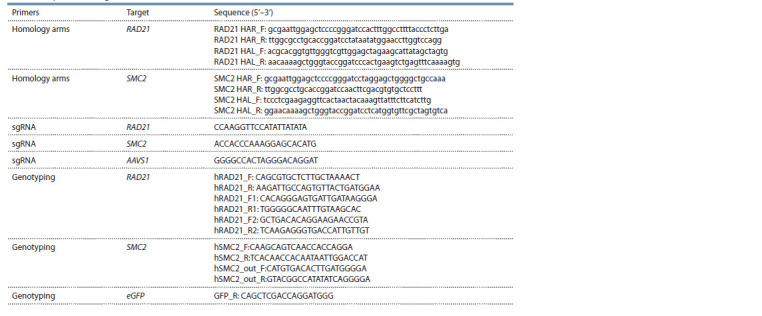
List of used primers and sgRNAs

Generation of HAP1 cell lines with an auxin-inducible degron system. Generation of degron cell lines was done essentially as previously described (Yunusova et al., 2021). Briefly, HAP1 cells were electroporated at conditions of 1200 V, 30 ms, 1 pulse, using the Neon Transfection system (Thermo Fisher Scientific), according to the manufacturer’s instructions with minor modifications. Per electroporation, 250,000 cells were resuspended in 10 μL of DPBS containing 1 μg of the plasmids with the ratio 1:1:2 (gRNA:Cas9:Donor vector for recombination, accordingly). Immediately following pulsation, cells were transferred into pre-warmed cell media without antibiotics. The next day cells were split into 10 cm dishes at 1:4 and 1:10 dilutions and placed under Hygromycin B selection (0.8 mg/ml) or puromycin selection (1 μg/ ml). The medium was replaced every three days. After 10 to 14 days of selection, single-cell clones were visible, and a subset of clones was handpicked with pipette tips under microscope.

Then part of the cells were lysed in PBND lysis buffer (0.2 mg/ml proteinase K, 10 mM Tris-HCl pH 8, 50 mM KCl, 2.5 mM MgCl2, 0.45 % (v/v) NP-40, and 0.45 % (v/v) Tween 20) or DNA extraction buffer (0.2 mg/ml proteinase K, 10 mM TrisHCl pH 8, 100 mM NaCl, 25 mM EDTA-Na2, 0.5 % SDS) for 1 h at 55 °C followed by proteinase K inactivation for 10 min at 95 °C. The target regions were amplified by PCR with HS-Taq DNA Polymerase (Biolabmix, Russia). The parameters were as follows: 95 °C for 30 s, then 34 cycles of 95 °C for 10 s, 60 °C (unless otherwise stated) for 30 s, 72 °C for 1 min/kb, and a final step at 72 °C for 5 min. The amplified products were analyzed by agarose gel electrophoresis

Protein detection. The cells were washed twice with PBS and scraped from the surface in the presence of RIPA buffer (50 mM Tris-HCl pH 8, 150 mM NaCl, 1 % Triton X-100, 0.5 % sodium deoxycholate, and 0.1 % SDS) containing the protease inhibitor cocktail (1x Complete ULTRA, 1x Phos- STOP (both from Roche, Switzerland), 5 mM NaF (Sigma- Aldrich)). After that, the cells were sonicated by three 10 s pulses at 33–35 % power settings with UW 2070 (Bandelin Electronics, Germany). Lysates were centrifuged at 14,000 g for 20 min at 2 °C, frozen, and stored at –80 °C. The protein concentrations in cell lysates were quantified using Pierce BCA Protein Assay Kit (Thermo Fisher Scientific). Equal amounts (20 μg) of total protein were separated on 10 % SDS-PAGE and then transferred onto the Immun-Blot PVDF membrane (Bio-Rad, USA). After blocking in 5 % milk/TBST for 2 h, the membrane was incubated with primary antibodies against RAD21 and SMC2 (#12673/#8720, Cell Signaling Technology, USA) at 4 °C overnight. On the following day, membranes were incubated with horseradish peroxidase–conjugated secondary antibodies (#7074, Cell Signaling Technology) for 2 h at room temperature. Detection was performed with Clarity™ (Bio-RAD) and detected iBright™ FL1500 (Thermo Fisher Scientific).

Chromosome spread. Chromosome preparations were made according to the previously described protocol with minor modifications (Kruglova et al., 2008). Briefly, human cell cultures were exposed to 50 ng/ml Colcemid (Merck KGaA, Darmstadt, Germany) for 3 h followed by auxin treatment for 2 hours. Afterwards, cells were detached with 0.05 % Trypsin-EDTA solution (Capricorn Scientific GmbH, Germany) and resuspended in hypotonic solution (0.38 M KCl) for 15 min at 37 °С. Then, cells were fixed with Carnoy fixative (3:1 methanol: glacial acetic acid), dropped onto cold wet glass slides, and stained with 1 μg/ml 4′,6-diamidino- 2-phenylindole (DAPI) (Sigma-Aldrich). The samples were analyzed using a Carl Zeiss Axioscop 2 fluorescence microscope at the Center for Collective Use of Microscopy of the Institute of Cytology and Genetics SB RAS (Novosibirsk, Russia). Image processing was carried out using ISIS software (MetaSystems GmbH, Germany). At least 50 metaphase plates were analyzed for each experimental group. Three categories of metaphase plates were distinguished: metaphases with separated chromatids, metaphases with non-separated chromatids, and an intermediate category of metaphase plates in which the chromatids either lay parallel, in close proximity, but were not in contact, or some of the chromosomes were with non-separated chromatids.

## Results

Introduction of modifications into the genome of cells In this study, we implemented a clone selection system based on chromosome morphology to obtain HAP1 cells capable of inducible depletion of RAD21 (a component of the cohesin complex) and SMC2 (a component of the condensin I and II complexes).

**Fig. 1.**
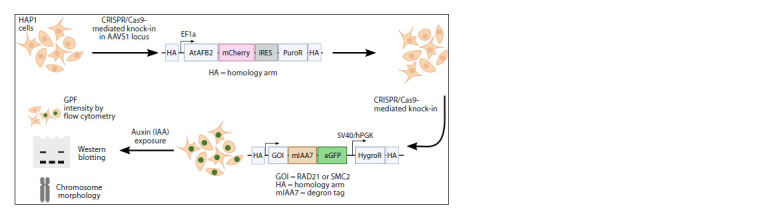
Overview of experimental design

To generate these cells, we employed the methodology described in the study by (Yunusova et al., 2021). The process of obtaining cell lines with auxin-dependent degradation of the protein of interest involved two rounds of genome modification, as illustrated in Fig. 1. Firstly, we performed targeted integration of an exogenous construct that encodes a degron fused with the eGFP gene and a selectable marker in front of the stop codon of the gene of interest. Secondly, we introduced the AtAFB2 gene, a component of plant ubiquitin ligase and an auxin receptor, into the cell genome through either random or targeted integration. In fact, modification of the gene of interest and integration of ubiquitin ligase AtAFB2 can be carried out in the reverse order, since effective operation of the system occurs only in cells that have both modifications.

In the presence of auxin, the AtAFB2 protein interacts with the degron domain, leading to polyubiquitination and subsequent degradation of the chimeric protein in the proteasome. This system allows for the controlled depletion of the protein of interest upon auxin induction.

PCR genotyping of cell clones with a degron

It is crucial to modify all alleles of the gene of interest to prevent residual wild-type alleles from maintaining protein function. For this study, we selected the human cell line HAP1 as the experimental cell line. The near haploid karyotype of HAP1 cells simplifies the process of obtaining modified subclones, as modification of a single allele of the gene of interest is sufficient. However, the presence of pseudogenes poses a significant challenge in clone selection, as they can complicate the identification of clones with the desired modification

**Fig. 2.**
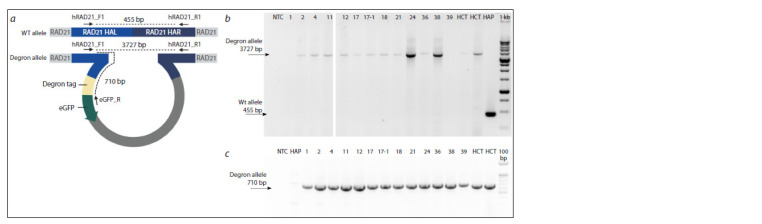
PCR genotyping of knockin in the RAD21 gene. a – scheme of primer annealing for the wild-type allele and the knockin allele; b – results of genotyping with primers hRAD21_F1-hRAD21_R1. It can be seen that none of the 13 cell clones tested contain the wild-type allele; c – genotyping results with primers hRAD21_F1-GFP_R. It can be seen that all 13 cell clones tested have the knockin allele. 1, 2, 4, 11, 12, 17, 17-1, 18, 21, 24, 36-1, 38, 39 – numbers of the tested clones; HCT – HCT116 RAD21_mAC; HAP – HAP1 without modifications; NTC – no template control; 1 kb and 100 bp ladders.

One such pseudogene, RAD21P1, located on the X chromosome, shares a high degree of homology (over 93 %) with the C-terminal fragment of the RAD21 gene (Supplementary Material 1)1. It is in this region that we inserted an exogenous construct containing a degron tag, eGFP, and a selectable marker (see Fig. 1). Therefore, the selection of appropriate primers for PCR genotyping is a critical step in the workflow. We carefully selected and tested several pairs of primers, as well as their combinations (see the Table), using DNA isolated from the HCT116 RAD21_mAC cell line as a positive con-trol sample. These cells were obtained and intensively characterized by another group (Yesbolatova et al., 2020), but since the design of the modification of the endogenous RAD21 gene was similar to what we used, these cells can be used as a reference for interpreting our results. Intact wild-type human DNA and DNA from one of the clones with targeted modification of the RAD21 locus, obtained in our laboratory, were also used as test samples. Indeed, some primer combinations amplified a nonspecific PCR product from the pseudogene even in the absence of a wild-type allele. Therefore, to genotype the selected clones, we used the hRAD21_F1/hRAD21_R1 primer combination (Fig. 2, a), which amplified only a specific product. PCR genotyping of clones with modification of the SMC2 gene did not reveal amplification of nonspecific fragments.


Supplementary Materials are available in the online version of the paper: https://vavilovj-icg.ru/download/pict-2024-28/appx7.pdf


At the first stage, the AtAFB2 gene was integrated into the AAVS1 safe-harbor locus, and puromycin-resistant cells were selected. These cells were then used for the second round of modification – insertion – of a construct with a degron at the end of the RAD21 gene. After selection on hygromycin, we selected more than a hundred colonies and performed PCR genotyping with primers hRAD21_F1/hRAD21_R1. Using the selected primers (see Fig. 2, a), we genotyped cell clones that had successfully passed selection for resistance to the antibiotic hygromycin. Figure 2, b, c shows the results of genotyping of 13 selected cell clones carrying the degron tag modification in the RAD21 gene.

The same genotyping strategy was used to establish HAP1 cell lines with the SMC2 degron (Supplementary Material 2).

Assessment of the degree of protein depletion upon induction of degradation by auxin

**Fig. 3.**
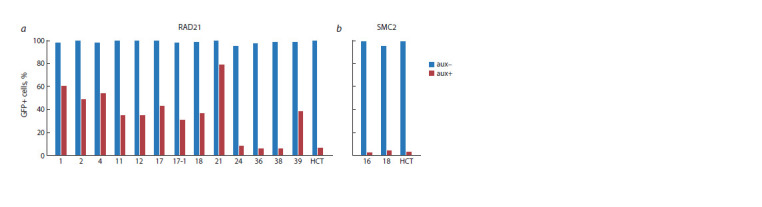
Evaluation of degradation efficiency of a protein of interest using flow cytometry. a – the proportion of cells that have not degraded (GFP+) fusion protein RAD21_miniIAA7_eGFP. 1, 2, 4, 11, 12, 17, 17-1, 18, 21, 24, 36, 38, 39 – numbers of tested clones; HCT – positive control, cells with efficient degradation of the protein of interest HCT116 RAD21_mAC; b – the proportion of cells that have not degraded (GFP+) fusion protein SMC2_miniIAA7_eGFP. 16, 18 – numbers of tested clones; HCT – positive control, cells with efficient degradation of the protein of interest HCT116 SMC2_mAC.

The degree of degradation of the chimeric protein RAD21_ miniIAA7_eGFP was assessed by the number of GFP-positive cells on a flow cytometer after 2 hours of exposure to auxin. Most of the clones demonstrated low efficiency of degradation of the target protein (Fig. 3, a). We assume that this is due to low expression in cells of the auxin receptor AtAFB2, which is integrated into the AAVS1 locus. Since we used AtAFB2 fused to the fluorescent protein mCherry (see Fig. 1), its expression level can be detected using a flow cytometer. Indeed, the level of mCherry fluorescence was higher in clones with more efficient degradation of RAD21_miniIAA7_eGFP (data not shown). We believe that integration of AtAFB2 transgene into a random location in the genome rather than into the AAVS1 locus is a better strategy, since in this case it is possible to select for clones in which the insertion provides strong, persistent expression of AtAFB2. This might happen due to the selection of clones with a high copy number of the insertion or with a successful epigenetic landscape at the site of integration. Based on this analysis, we continued to functionally characterize the depletion efficiency of only those clones that showed a degree of degradation comparable to the HCT116 RAD21_mAC cell line (Yesbolatova et al., 2020).

A similar analysis was carried out for subclones with the chimeric protein SMC2_miniIAA7_eGFP. In this case, both subclones tested had a degree of chimeric protein degradation comparable to the control sample (SMC2_mAID_Clover) (see Fig. 3, b). SMC2_mAID_Clover cells were previously obtained using a similar strategy by tagging the SMC2 gene in (Yesbolatova et al., 2020).

Selected cell clones were assessed for loss of protein function by analyzing metaphase chromosome morphology.

Assessment of loss of function of cohesin and condensins based on chromosome morphology analysis

**Fig. 4.**
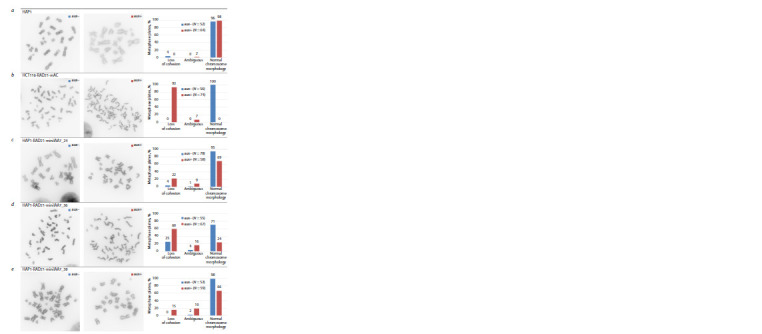
Assessment of changes in chromosome morphology after induction of RAD21 depletion. For each cell clone, one metaphase plate is presented, illustrating the most common chromosome morphology without induction (aux–) and after induction (aux+). Also presented is a diagram summarizing the proportion of metaphase plates with loss of chromatid cohesion, normal chromosome morphology and ambiguous. For each condition, the number of analyzed metaphase plates is indicated – N. a – HAP1 cells without modifications – negative control; b – HCT116 RAD21_mAC, cells with effective RAD21 depletion – positive control; c–e – cell clones tested.

**Fig. 5.**
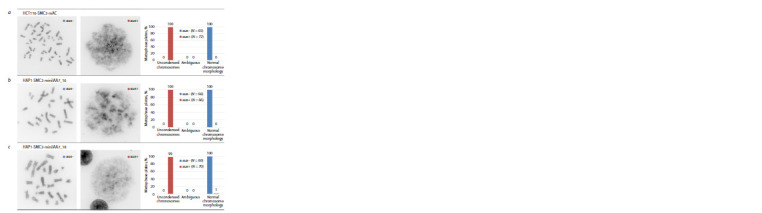
Assessment of changes in chromosome morphology after induction of SMC2 depletion. For each cell clone, one metaphase plate is presented, illustrating the most common chromosome morphology without induction (aux–) and after induction (aux+). Also presented is a diagram summarizing the proportion of metaphase plates with uncondensed chromosomes, normal chromosome morphology and ambiguous. For each condition, the number of analyzed metaphase plates is indicated – N. a – HCT116-SMC2-mAC; cells with effective SMC2 depletion – positive control; b, c – cell clones tested.

Since the architectural proteins of chromatin – the cohesin and condensin complexes – have a well known function in the formation of mitotic chromosomes, changes in chromosome morphology can serve as a convenient criterion for assessing the function of these proteins. Therefore, we assessed the proportion of cells with abnormal chromosome morphology in clones before and after depletion of the target protein induced by auxin exposure. Cohesin ensures cohesion of sister chromatids; therefore, in clones with RAD21_miniIAA7_ eGFP, we counted the number of metaphase plates with unconnected chromatids (Fig. 4). Condensins are responsible for the compaction of metaphase chromosomes and the formation of their rod-shaped morphology; therefore, for clones with SMC2_miniIAA7_eGFP, we counted the number of metaphase plates with non-compacted chromosomes (Fig. 5).

It is clearly seen from Fig. 4 that in unmodified cells, cohesion of sister chromatids is observed in the vast majority of metaphase plates (see Fig. 4, a). We use HCT116 RAD21_mAC cells as a positive control, since these cells demonstrate effective depletion of RAD21 (Yesbolatova et al., 2020). For these cells, without auxin exposure, all plates have chromosomes with sister chromatid cohesion. And when depletion of RAD21 is induced by auxin, no plates with normal chromosome morphology remain, and the vast majority of plates contain separate chromatids (see Fig. 4, b). This shows that the chromosome morphology test allows us to assess cohesin function both before and after induction of RAD21 depletion by auxin. None of the three HAP1 cell clones (see Fig. 4, c–e) showed loss of cohesin function comparable to the positive control. In clones 24 and 38, there is no loss of chromatid cohesion in most laminae. And in clone 36, even in the absence of auxin induction, 25 percent of the plates did not have chromatid cohesion, that is, cohesin function was impaired without induction.

In the case of the SMC2 degron, both selected clones showed the same complete loss of condensin function after induction as the control and no evidence of protein function deficiency without auxin induction (see Fig 5).

Evaluation of cohesin and condensins depletion based on Western blotting

Assessing the efficiency of degradation of a protein of interest based on flow cytometry is indirect, since in this way only the protein fused with degron and fluorescent domains will be included in the analysis. It is possible to propose several scenarios in which, despite the degradation of the fusion protein, functional molecules of the protein of interest remain in the cells (we discuss these scenarios in Discussion). Therefore, it is important to complement the analysis of the morphology of metaphase chromosomes with the Western blotting methods

**Fig. 6.**
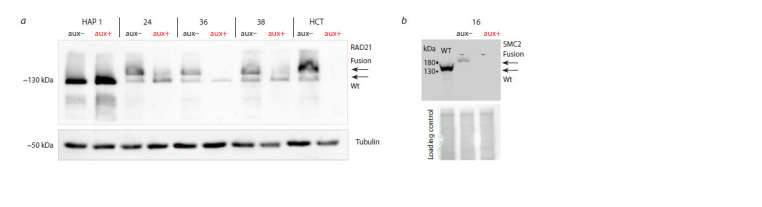
Identification of proteins of interest using Western blotting. a – Western blotting of RAD21 in cells without modification (HAP), in cells with effective depletion of RAD21 (HCT) and three cell clones tested (24, 36, 38) without induction (aux–) and after induction of depletion (aux+). For RAD21, the sizes of the fusion protein and the wild-type protein are indicated. Tubulin detection was used as an internal control; b – Western blotting of SMC2 in cells without modification (wt), and cell clone tested (16) without induction (aux–) and after induction of depletion (aux+). Total protein staining (loading control).

Figure 6, a shows the results of Western blotting of the RAD21 protein. It is clearly seen that the amount of RAD21 protein in unmodified cells is greater than in modified cells. In positive control cells HCT116 RAD21_mAC after induction with auxin, RAD21 is not detected at all. In clones 24, 36, 38, two forms of the protein are detected, a heavier one corresponding to the fusion protein with GFP and a lighter one corresponding to the wild-type protein. After exposure to auxin, fusion protein decreases (clones 24, 38) or completely disappears (clone 36). However, in clones 24, 36, 38, the wild-type form of the RAD21 protein does not decrease after auxin induction.

Western blotting of the SMC2 protein carried out for clone 16 also showed that knockin of the degron domain into the SMC2 gene leads to a decrease in the amount of protein compared to unmodified cells. After induction of protein degradation by auxin, SMC2 is not detected (see Fig. 6, b).

## Discussion

Protein function studies are often based on the loss of function strategy. However, for many proteins, loss of function will have a dramatic impact on viability. The use of inducible depletion systems makes it possible to apply a loss of function approach to such proteins. However, like any complex technology, inducible protein depletion has its own limitations. In this article, we described an approach based on the use of chromosome morphology as a convenient functional test for selecting clones with inducible depletion of the architectural chromatin proteins cohesin and condensins.

The difficulty of obtaining cell clones capable of inducible protein depletion appears to depend greatly on the properties of the particular protein of interest. In our work, clones capable of effective loss of SMC2 function were obtained easily, without any trouble at the stage of genotyping the clones or at subsequent stages of assessing the completeness of degradation. At the same time, the attempt to obtain a clone capable of efficient degradation of the RAD21 protein was not successful: the clones we obtained suffer from loss of protein function without auxin induction and incomplete protein degradation after induction. This is clearly visible when comparing these parameters with HCT116 RAD21_mAC cells, which we chose as a positive control (these cells are capable of effective auxininduced depletion of RAD21) (Yesbolatova et al., 2020).

We believe that in the case of RAD21, the main problem is the decrease in the amount of RAD21 in cells even in the absence of auxin induction (“basal degradation”). This decrease may have two independent causes: 1) instability of the transcript/protein due to fusion with the degron tag and GFP. Theoretically, introduction of a genetic construct could reduce the knockin transcription efficiency of RAD21; cause a decrease in transcript stability; reduce the efficiency of translation of the knockin transcript; cause a decrease in the stability of the fusion protein (Yu et al., 2015); 2) background ubiquitin ligase activity in the absence of auxin. Such activity will lead to polyubiquitinylation and degradation of the protein carrying the degron tag (Li et al., 2019). Apparently, both occur in our case. To address the first cause, one can do little except make a fusion not with the C-terminus, but with the N-terminus of the peptide. But the second cause depends entirely on the properties of the peptide used and the auxin-dependent ubiquitin ligase. We used the ubiquitin ligase AtAFB2, which is thought to have lower basal activity compared to OsTIR1 (Li et al., 2019). However, a system with even lower activity has recently emerged. It was created based on OsTIR1 by replacing the ligand with a synthetic auxin analogue 5-Ph-IAA and rationally designing the active site of the enzyme (Yesbolatova et al., 2020). Our positive control cells are designed with just such a system. The Western blot in Fig. 6 clearly shows that the amount of the heavy fusion form of the protein in the positive control is significantly greater than in clones 24, 36, 38 that we created based on AtAFB2. This may be explained by the higher basal activity of AtAFB2. In addition, the use of alternative depletion systems, such as dTAG, may be a productive strategy for achieving degradation of architectural chromatin proteins (Nabet et al., 2018).

As we noted above, the key stage in obtaining cells capable of inducible depletion is the complete modification of all alleles of the gene of interest. All cell clones used in this work did not have wild-type alleles when analyzed by PCR (see Fig. 2). However, Western blotting reveals a band corresponding in size to unmodified RAD21. Since there are no wild-type alleles during genotyping, this eliminates the possibility of contamination of samples with unmodified cells. Theoretically, repair of the break introduced by Cas9 can lead to the loss of the primer annealing site (we previously reported this issue (Korablev et al., 2020)); such damaged alleles will not be detected by PCR, however, they can produce a functional transcript. But for haploid HAP1 cells, this explanation does not apply since each clone contains exactly one modified RAD21 allele. Alternative explanations suggest that wild-type RAD21 appears in cells containing the correct modification of RAD21. For example, a transcript from a modified allele can be spliced and, as a result, a fusion peptide is not formed. During translation, a peptide bond may also not be formed, for example, like in 2A viral peptides. One can speculate that something similar may form between the fusion parts of the peptide. However, both of these explanations in our case remain at the level of speculation.

For some genes, pseudogenes could be the source of a functional peptide (Zhang et al., 2023); however, in the case of RAD21, the pseudogene is highly mutated and contains many stop codons and therefore does not have a functional open reading frame

Of course, the most direct way to assess depletion of a protein of interest is Western blotting, but this method is highly dependent on the quality and specificity of the antibodies. It is known that the specificity of many antibodies is questioned (Baker, 2015).

## Conclusion

Therefore, for many proteins, the use of Western blotting can be problematic. Therefore, a convenient functional test is an excellent way to characterize cells capable of induced depletion of a protein of interest. The chromosome morphology test used in this work proved to be very informative.

## Conflict of interest

The authors declare no conflict of interest.
